# Cloning and Characterization of Farnesyl Diphosphate Synthase Gene Involved in Triterpenoids Biosynthesis from *Poria cocos*

**DOI:** 10.3390/ijms151222188

**Published:** 2014-12-02

**Authors:** Jianrong Wang, Yangyuan Li, Danni Liu

**Affiliations:** Guangdong VTR Bio-Tech Co., Ltd., Zhuhai 519060, Guangdong, China; E-Mails: liyangyuanvtr@163.com (Y.L.); danni_vtr@163.com (D.L.)

**Keywords:** farnesyl diphosphate synthase, methyl jasmonate, *Poria cocos*, triterpenoids

## Abstract

*Poria cocos* (*P. cocos*) has long been used as traditional Chinese medicine and triterpenoids are the most important pharmacologically active constituents of this fungus. Farnesyl pyrophosphate synthase (FPS) is a key enzyme of triterpenoids biosynthesis. The gene encoding FPS was cloned from *P. cocos* by degenerate PCR, inverse PCR and cassette PCR. The open reading frame of the gene is 1086 bp in length, corresponding to a predicted polypeptide of 361 amino acid residues with a molecular weight of 41.2 kDa. Comparison of the *P. cocos* FPS deduced amino acid sequence with other species showed the highest identity with *Ganoderma lucidum* (74%). The predicted *P. cocos* FPS shares at least four conserved regions involved in the enzymatic activity with the FPSs of varied species. The recombinant protein was expressed in *Pichia pastoris* and purified. Gas chromatography analysis showed that the recombinant FPS could catalyze the formation of farnesyl diphosphate (FPP) from geranyl diphosphate (GPP) and isopentenyl diphosphate (IPP). Furthermore, the expression profile of the FPS gene and content of total triterpenoids under different stages of development and methyl jasmonate treatments were determined. The results indicated that there is a positive correlation between the activity of FPS and the amount of total triterpenoids produced in *P. cocos*.

## 1. Introduction

*Poria cocos* (*P. cocos*) has been used as traditional medication for several thousand years in East Asia. The predominant triterpenoids in *P. cocos* are pachymic acid and eburicoic acid. Previous studies have shown that triterpenoids separated from this fungus had a number of important pharmacological activities. Pachymic acid and dehydrotumulosic acid isolated from *P. cocos* actively inhibited phospholipase A2 from snake venom [1]. Tai et al (1995) [2] demonstrated that pachymic acid extracted from *P. cocos* with a methylene group at C-24 exhibited antiemetic activity in frogs. Six lanostane-type triterpene acids isolated from *P. cocos* showed cytotoxic activity against some human cancer cells [3,4]. However, despite the potential medical importance, little is known about the molecular biology of triterpenoid biosynthesis in *P. cocos*. 

**Figure 1:**
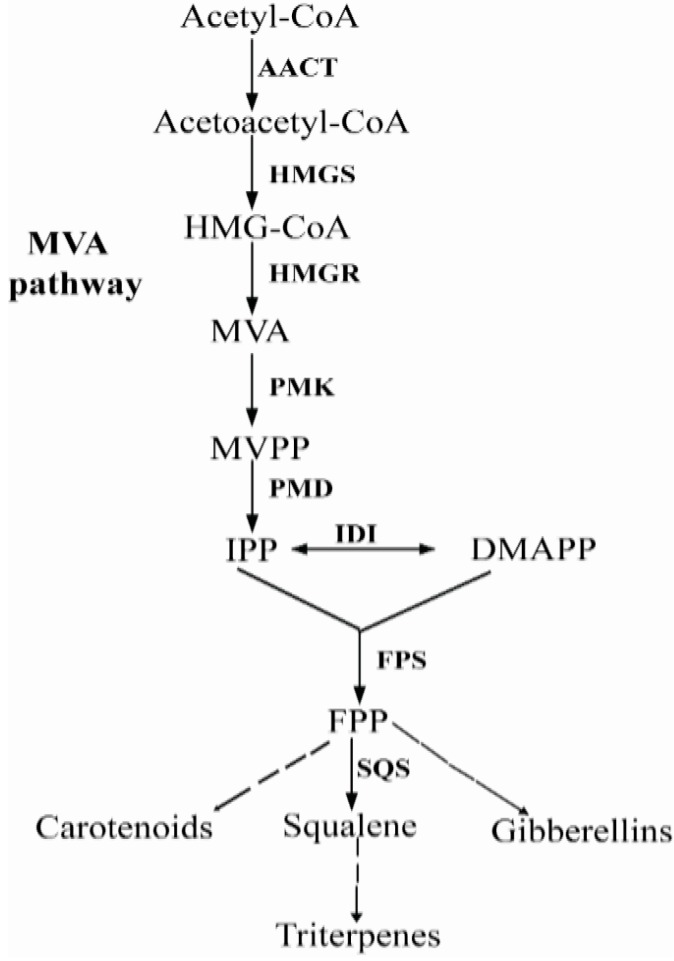
Main steps in the biosynthesis of terpenoids in fungi. The abbreviations used are: HMG-CoA: hydroxymethylglutaryl CoA; MVP: 5-phosphomevalonate; MVPP: 5-diphosphomevalonate; IPP: isopentenyl pyrophosphate; DMAPP: dimethylallyl pyrophosphate; FPP: farnesyl pyrophosphate. Enzymes are indicated in bold; AACT: acetoacetyl-CoA thiolase; HMGS: HMG-CoA synthase; HMGR: HMG-CoA reductase; MVK: mevalonate kinase; PMK: phosphomevalonate kinase; PMD: mevalonate 5-diphosphate decarboxylase; IDI: IPP isomerase; FPS: FPP synthase; SQS: Squalene synthase.

Triterpenoids are synthesized via the isoprenoid pathway. The isoprenoid pathway constitutes the most diverse and widespread metabolic pathway of all prokaryotes and eukaryotes, resulting in the biosynthesis of a large number of primary as well as secondary metabolites. Isoprenoids are derived from common C_5_ building blocks namely IPP and its isomer dimethylallyl diphosphate (DMAPP). IPP and DMAPP are synthesized via two independent and non-homologous pathways, the MVA (mevalonate) and MEP (2-C-methyl-d-erythritol 4-phosphate) pathways [5,6]. In plants and algae, isoprenoids are formed via both the MVA and MEP pathways [7]. In fungi, however, isoprenoids are synthesized through the classical MVA pathway. Different isoprenoids in fungi share all the early intermediates, up to FPP through the MVA pathway, which is catalyzed by acetoacetyl-CoA thiolase (AACT), 3-hydroxy-3-methylglutaryl-CoA synthase (HMGS), HMG-CoA reductase (HMGR), MVA kinase (MVK), phosphor mevalonate kinase (PMK), MVA-5-diphosphate decarboxylase (PMD), isopentenyl diphosphate isomerase (IDI) and farnesyl diphosphate synthase (FPS) (Figure 1) [8,9]. As shown in Figure 1, FPS is a key enzyme in terpenoids biosynthesis, which catalyzes the consecutive condensations of dimethylallyl diphosphate (DMAPP) or GPP with IPP to produce FPP [10]. FPP is located at the ﬁrst multiple branch point in the isoprenoid biosynthetic pathway for several terpenoids, like triterpenes, carotenoids, gibberellins and sterols [11].

Upon until now, the complementary DNA (cDNA) or genomic DNA encoding FPS have been cloned and characterized from yeast, fungi, higher plants and human [10,11,12]. These studies have demonstrated that the FPS gene (*fps*) size varies from 1.2 to 2.1 kb. In plants, *fps* belongs to a small multigenic family that encodes at least two different isoforms. *Arabidopsis thaliana* contains two *fps* genes, which encode three FPS isoforms [13]. Three FPS isoforms have also been discovered in both maize and *Artemisia tridentate* [14,15]. In human, only a single *fps* encodes for FPS. Due to the alternative splicing in the first exon of human *fps,* multiple splice variants are generated which encode two FPS isoforms: a shorter cytoplasmic/peroxisomal form, and a longer isoform which is a mitochondrial targeting peptide [16]. 

So far, there are no reports about gene cloning and characterization of *fps* from *P. cocos*. For a better understanding of the role of FPS in triterpenoids biosynthesis in *P. cocos*, we firstly needed to know the *fps* and its expression profile in *P. cocos*; thus, we here describe the cloning and characterization of *fps* from *P. cocos*. Further, the expression profile of *P. cocos* FPS gene (*Pc-fps*), and content of total triterpenoids at different stages of development and methyl jasmonate treatments in *P. cocos* were also investigated. Our results should be helpful for further study of its structure and expression, and facilitate the investigation of the role of FPS in the control of triterpenoids biosynthesis in *P. cocos*. 

## 2. Results and Discussion

### 2.1. Isolation and Sequence Analysis of Pc-fps

A 480 bp putative fragment of the *Pc-fps* was obtained by degenerate PCR. To obtain the 5'- and 3'-flanking regions of the putative *Pc-fps*, inverse PCR and cassette PCR were conducted with oligonucleotide primers specific for the partial sequences of *Pc-fps*. Sequence analysis showed that the cloned genomic DNA is 3120 bp in length, including the 5'-untranslated region (UTR) of 738 bp, 3'-UTR of 725 bp, five exons, and four introns*.* The genomic sequence of *Pc-fps* consisted of 1767 bp and RT-PCR strategies were applied to isolate the putative cDNA of *Pc-fps*. The open reading frame of *fps* is 1086 bp in length and corresponded to a predicted polypeptide of 361 amino acid residues, with a predicted molecular weight of 41.29 kDa and pI of 5.40. The nucleotide sequences of the *Pc-fps* and *Pc-fps* cDNA have been deposited in the GenBank database, with accession numbers JN652835 and JN652836, respectively.

### 2.2. Sequence Features of the Pc-fps Promoter Region

**Figure 2:**
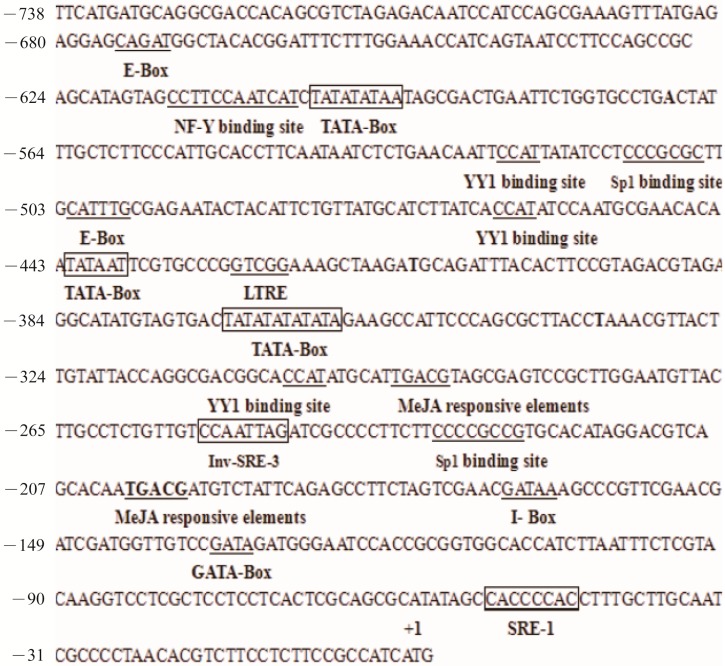
Nucleotide sequence of the promoter region for the *Pc-fps* promoter. The translational start site ATG was defined as position +1. The transcriptional regulatory elements of TATA-box, Sp1 binding site, NF-Y binding site, E-box motif, YY1 binding site and MeJA responsive elements are labeled and underlined.

Bioinformatics analysis revealed that the *Pc-fps* promoter carried typical eukaryotic promoter elements (Figure 2). Three predicted TATA boxes were found at position −567, −410 and −367 with respect to the ATG codon, as well as 10 putative CAAT boxes. Three possible core promoter regions were found at −607 to −558, −450 to −401 and −373 to −324 and scores from 0.98 to 1.0 were predicted by using the NNPP v.2.2 for promoter prediction. Potential regulatory elements associated with sterol biosynthesis, hormone, and stress-related responses were also found in the *Pc-fps* promoter region; there was a Sp1 binding site, an NF-Y binding site, and an E-box motif. Some regulatory elements were homologous to the consensus elements (Inv-SRE-3, SRE-1) of the human squalene synthase promoter [17]. Two methyl jasmonate (MeJA) responsive elements (TGACG) were found at position −235 to −230 and −152 to −147. Analysis of these specific motifs of the *Pc-fps* promoter region might identify useful cis-acting elements and thus provide some clues to the mechanism by which this gene responds to stress signals.

### 2.3. Comparison of FPS Protein Sequences

**Figure 3:**
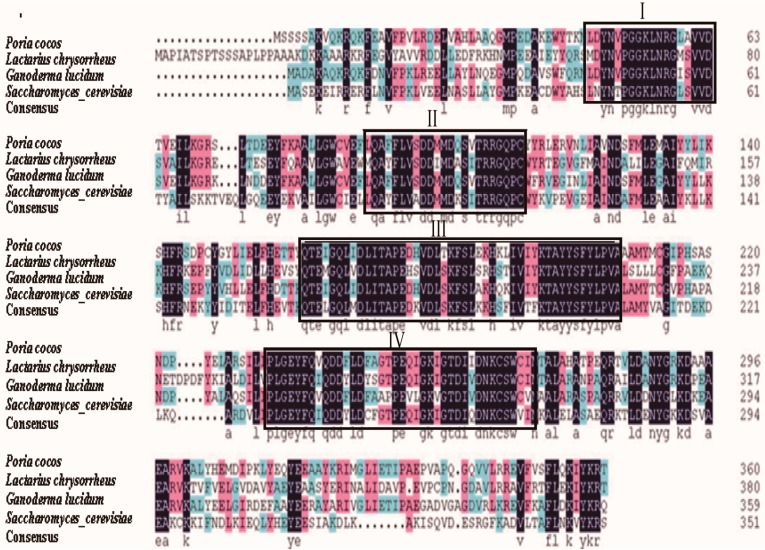
Sequences of other fungal species were obtained from the GenBank (*Ganoderma lucidum*, GenBank number: ACB37020; *Saccharomyces cerevisiae*, GenBank number: EGA61811.1; *Lactarius chrysorrheus*, GenBank number: BAD15361.1). Four highly similar regions (I, II, III and IV) were boxed.

The NCBI protein-protein BLAST showed that the deduced *P. cocos* FPS *(*Pc-FPS*)* amino acid sequence shared 74% identity with the FPS of *G. lucidum* (GenBank number: ACB37020). The identity level was also high with other fungi, such as *S. cerevisiae* (GenBank number: EGA61811.1), *Lactarius chrysorrheus* (GenBank number: BAD15361.1), whereas Pc-FPS shared a relatively lower identity to the FPSs of animals and plants (data not show). Alignment of Pc-FPS with other fungal FPSs displayed at least four conserved regions, designated I to IV (Figure 3). The conserved GGKXXR motif was present in region I. The highly conserved aspartate-rich motif (DDXX(XX)D) was present in regions II and IV, which are perhaps involved in substrate binding through the formation of magnesium salt bridges between the pyrophosphate moieties of the isoprenoid substrate and the carboxyl group of the aspartate [18]. In addition, Pc-FPS possesses two aromatic residues (FF) at the fifth and fourth positions before the first aspartate-rich motif, which are involved in the product chain-length. This implies that Pc-FPS has an FPS activity that does not yield long-chain products [19]. We have also identified a highly conserved GGKXXR motif in the region I; the presence of arginine and lysine in region I, indicates probable involvement in substrate binding [20].

### 2.4. Phylogenetic Analysis

**Figure 4:**
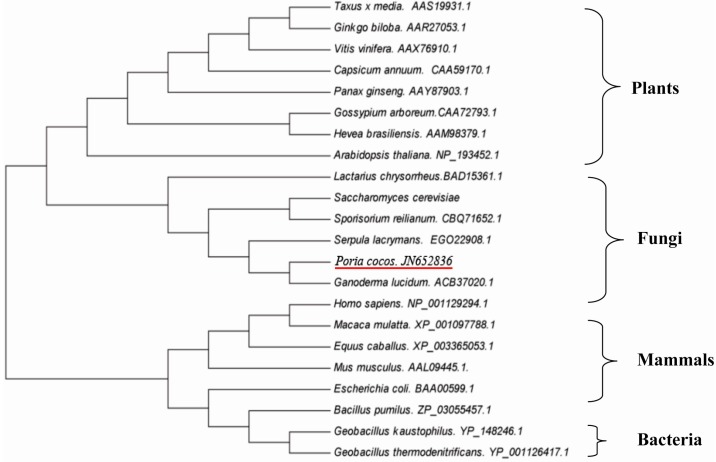
Phylogenetic analysis of FPS amino acid sequences using a neighbor-joining (NJ) method. Twenty-two sequences from different species were retrieved from GenBank.

A phylogenetic tree was constructed using known FPS amino acid sequence from a wide rang of different organisms including plants, animals, bacteria and fungi. The fungal enzymes were clustered into one group, in which the FPS of *P. cocos* was more closely related to the FPS of *G. lucidum* than to those of other fungi and they formed a distinct subgroup (Figure 4).

### 2.5. Expression of Pc-FPS in Pichia pastoris (P. pastoris)

**Figure 5:**
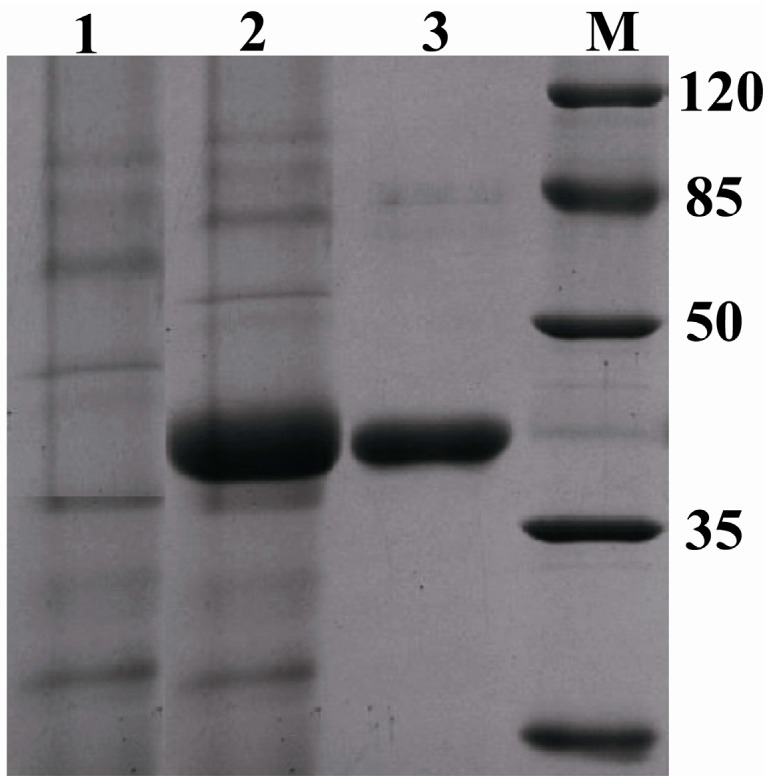
SDS-PAGE analysis of culture supernatant and purified rPc-FPS. Lane **M**: molecular mass marker; lane **1**: culture supernatant from mock transformant *P. pastoris* X33 containing expression vector pPICZαA (negative control); lane **2**: culture supernatant culture supernatant from transformant *P. pastoris* X33 containing expression vector pPICZαA-*fps*; lane **3**: purified rPc-FPS.

A 1086-bp *fps* was integrated in frame with the *S. cerevisiae* α-factor secretion signal sequence under the control of the AOX1 promoter in pPICZαA to obtain the expression vector pPICZαA-*fps*. Plasmid pPICZαA-*fps* was transformed into *P. pastoris* X33. To select the FPS producing clones, we randomly picked 20 positive colonies and inoculated them into 500 mL shaking flasks containing 50 mL Buffered Glycerol-complex Medium (BMGY). SDS-PAGE was used to observe the expression of the recombinant protein (Figure 5). SDS-PAGE was carried out on a 12% running gel and stained with Coomassie Blue. As shown in Figure 5, the molecular weight of rPc-FPS was about 42 kDa, which was same as the calculated molecular weight. After purification by Ni^2+^-chelating chromatography, the purified rPc-FPS showed a single band on SDS–PAGE with molecular weight of 42 kDa (Figure 5).

### 2.6. Determination of FPP-Derived Farnesol by Gas Chromatography (GC)

**Figure 6:**
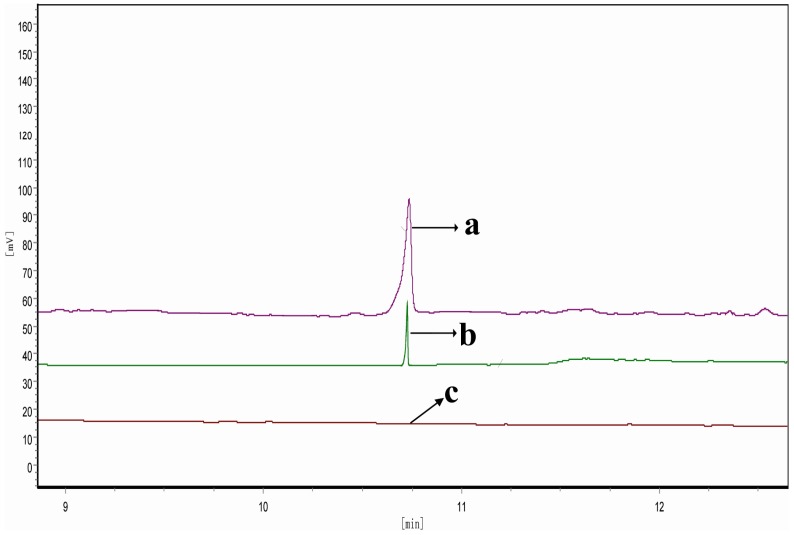
GC detection of the catalyzed product of the recombinant enzyme encoded by *Pc-fps*. (a) authentic FPP-derived farnesol; (b) the sample extracted from an *in vitro* reaction mixture containing the purified rPc-FPS; (c) The sample extracted from an *in vitro* reaction mixture containing the product from a mock purification scheme of mock transformant *P. pastoris* X33 containing expression vector pPICZαA.

To confirm the *Pc-fps* as a functional gene encoding FPS, the purified rPc-FPS was used to catalyze GPP with IPP to produce FPP. After a catalytic reaction at 30 °C for 3 h, the mixture was extracted with l-butanol saturated with water and then treated with potato acid phosphatase at 37 °C to hydrolyze the diphosphate moiety. The hydrolyzed products (FPP-derived farnesol) were extracted with hexane and analyzed by GC. Figure 6 shows the GC results. A comparison of the retention time of the samples with those of authentic FPP-derived farnesol (Figure 6a) confirms that the rPc-FPS could catalyze the formation of FPP from GPP and IPP (Figure 6b). By contrast, no such product peak was detected in the sample extracted from an *in vitro* reaction mixture containing the product from a mock purification scheme of mock transformant *P. pastoris* X33 containing expression vector pPICZαA (Figure 6c). These results indicate that the *fps* from *P. cocos* is a functional gene encoding FPS. 

### 2.7. Analysis of Expression Profile of Pc-fps and Total Triterpenoids at Different Stages of Development

**Figure 7:**
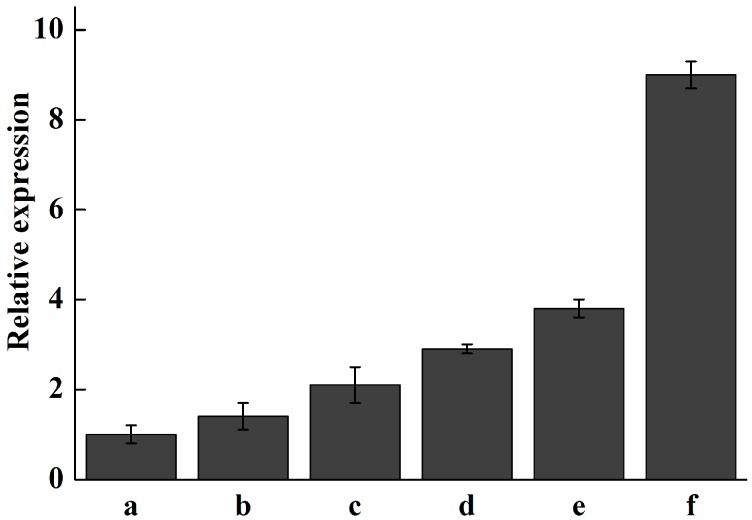
Analysis of expression levels of *Pc-fps* in *P. cocos* at various developmental stages. (a) 5-day-old mycelium; (b) 10-day-old mycelium; (c) 15-day-old mycelium; (d) 20-day-old mycelium; (e) 30-day-old mycelium; (f) primordia.

**Figure 8:**
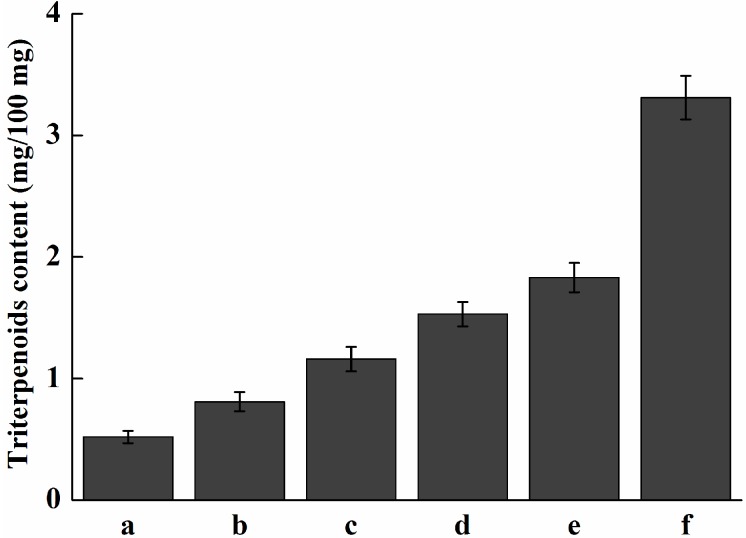
Analysis of the content of total triterpenoids in *P. cocos* at various developmental stages. (a) 5-day-old mycelium; (b) 10-day-old mycelium; (c) 15-day-old mycelium; (d) 20-day-old mycelium; (e) 30-day-old mycelium; (f) primordia.

The transcriptional level of *Pc-fps* at different stages of development was determined by real-time quantitative PCR. As shown in Figure 7, gene expression was relatively low in 5-to-15-day-old mycelia. However, the expression level was increased after 20-to-30-day-old mycelia. The fruiting process increased the expression level of *Pc-fps*. In the primordia, the expression level of *Pc-fps* was 9-fold higher than that of *Pc-fps* in the 5-day old mycelia. The content of total triterpenoids under different stages of development is shown in Figure 8. As shown in Figure 8, the content of total triterpenoids was relatively low in 5-to-15-day-old mycelia and increased gradually after 20-to-30-day-old mycelia. The content of total triterpenoids of primordia was 3.31 mg/100 mg, which was 1.8-fold higher than the 30-day-old mycelia (1.83 mg/100 mg). FPS as a key enzyme in triterpenoids biosynthesis and the activity of FPS may influence the amount of triterpenoids produced in *P. cocos*. In this study, we analyzed the expression profile of *Pc-fps* and content of total triterpenoids under different stages of development. The results showed that there is a positive correlation between the expression level of *Pc-fps* and the amount of total triterpenoids produced in *P. cocos.* During the 5-to-15-day-old mycelia, the expression level of *Pc-fps* and total triterpenoids content were relatively low. However, the expression level and total triterpenoids content were increased gradually after 20-to-30-day-old mycelia. During all different stages of development, the expression level and total triterpenoids content of primordia were higher than other stages.

### 2.8. Analysis of Expression Profile of Pc-fps and Content of Triterpenoids under Methyl Jasmonate (MeJA) Treatments

**Figure 9:**
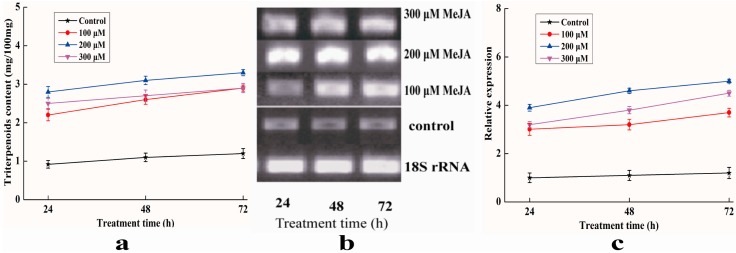
(**a**) Effects of different MeJA concentrations on the content of total triterpenoids in *P. cocos*; (**b**) RT-PCR analysis of expression level of *Pc-fps* treated with different concentrations of MeJA; (**c**) Real-time quantitative PCR analysis of expression level of *Pc-fps* treated with different concentrations of MeJA.

The effects of MeJA induction on expression profile of *Pc-fps* and content of total triterpenoids in *P. cocos* is shown in Figure 9. The results shown in Figure 9a indicate that there is a significant difference in total triterpenoids production between the control and experimental groups demonstrating that MeJA can potently stimulate triterpenoids biosynthesis. The total triterpenoids content increased from 2.11 to 3.21 mg/100 mg, from 2.68 to 3.63 mg/100 mg and from 2.47 to 3.31 mg/100 mg when MeJA concentrations were 100, 200 and 300 μM, respectively. The total triterpenoids content was approximately 3.16-fold higher than the control sample when the concentration of MeJA was 200 μM and the induction time was 72 h. Figure 9b shows the results of RT-PCR analysis of expression level of *Pc-fps* treated with different concentrations of MeJA. *Pc-fps* transcripts accumulated following the addition of 100–300 μM MeJA to the culture medium; the highest mRNA level was observed at a concentration of 200 μM MeJA and the induction time was 72 h. Furthermore, the transcriptional level of *Pc-fps* was also determined by real-time quantitative PCR. As shown in Figure 9c, transcription accumulation was obvious in the *Pc-fp*s expression under all MeJA stimulation conditions. The transcriptional level of *Pc-fps* was highly enhanced following the addition of 100–300 μM MeJA to the culture medium. The transcriptional level was approximately 4-fold higher than the control sample when the induction concentration was 200 μM and the induction time was 72 h. Sequence analysis of the *Pc-fps* promoter identified two potential MeJA responsive elements. MeJA are considered as potent lipid regulators that modulate various physiological processes in plants [21]. So for, MeJA has been used to induce or increase useful secondary metabolites [22,23]. However, there has been no such report on the effect of MeJA on the activities of the *fps* in *P. cocos*. In this study, we found that the MeJA treatment could significantly increase the transcription level of *Pc-fps* and content of total triterpenoids. Our results are consistent with previous reports, which demonstrated that MeJA could increase the biosynthesis of ganoderic acid as well as the transcript level of the *fps* in *G. lucidum* [24]. These results suggest that *Pc-fps* is probably involved in the triterpenoids biosynthesis regulation pathways of MeJA signalling via the putative MeJA-response elements. However, further studies are needed to define precisely and elucidate the *Pc-fps* promoter elements involved in the regulation of *P. cocos* triterpenoids biosynthesis. These results are useful to facilitate the investigation of the role of FPS in the control of triterpenoids biosynthesis in *P. cocos*. 

## 3. Experimental Section 

### 3.1. Cultures and Materials

*P. cocos* culture was purchased from the Institute of Applied Mycology of Huazhong Agricultural University. The *E. coli* strain DH5α is routinely conserved in our laboratory. *Pichia pastoris* (*P. Pastoris*) X-33, the expression vector pPICZαA and zeocin were purchased from Invitrogen (Carlsbad, CA, USA). Restriction enzymes, T4-DNA ligase and DNA polymerase were purchased from Sangon Biotech (Shanghai, China). The oligonucleotides were synthesized by the Shanghai Generay Company (Shanghai, China). GPP, IPP, FPP and methyl jasmonate were purchased from Sigma-Aldrich (St. Louis, MO, USA).

### 3.2. Cloning of Pc-fps

The integral sequence of the *Pc-fps* was acquired by degenerate PCR. Two degenerate primers Fs_1_ and Fs_2_ were designed on the basis of the highly conserved amino acid sequence PGGKLNKG and KTAYYSFY among *G. lucidum* and *Lactarius chrysorrheus*. Based on the partial sequence information, inverse PCR and cassette PCR were used to obtain the 5' flanking regions and the 3' flanking regions of the *Pc*-*fps*. To confirm the introns and obtain the full-length cDNA of *Pc*-*fps*, reverse transcription and PCR was conducted. Primers used in this study are listed in Supplementary Table S1.

### 3.3. Bioinformatics Analysis 

The cloned sequences were first analyzed via online BLASTn provided by National Center for Biotechnology Information (Bethesda, MD, USA). Analysis of the genome was performed by using Recognition of Regulatary Motifs with statistics in the softberry software (http://www.softberry.rn/berry.html) and PLACE Web Signal Scan (http://www.dna.affrc.go.jp/PLACE/signalscan.html).The promoter prediction was carried out using the Neural Network promoter prediction (NNPP) of the Berkeley Drosophila Genomic Project (BDGP; http://www.frustfly.org/seq-tools/promoter.html).The sequences were analyzed for identity using DNAman Version 6 (Lynnon Biosoft, San Ramon, CA, USA). Phylogenetic analysis of FPS amino acid sequences was made using the neighbor-joining (NJ) method by MEGA 4 [25]. 

### 3.4. Vector Construction and Transformation and Selection of P. pastoris Clones 

The *Pc-fps* was double digested with EcoRI and XbaI, and then ligated into pPICZαA, forming pPICZαA-*Pc-fps*. Finally, the expression vector pPICZαA-*Pc-fps* was used to transform *E. coli* Top 10. Through DNA sequencing, pPICZαA-*Pc-fps* was confirmed to contain the *Pc-fps*. *P. pastoris* X-33 transformed with 10 μg of pPICZαA and PmeI-linearized pPICZαA-*Pc-fps* vector by electro-transformation, according to Invitrogen’s recommendations. Transformants were plated on YPDS plates to isolate resistant clones. Transformed colonies were confirmed by both PCR and sequencing.

### 3.5. Shaking Flask Cultures and Purification of the Recombinant FPS (rFPS)

Transformed colonies confirmed by both PCR and sequencing were selected for shaking flask cultures. The transformed colonies contained pPICZαA was set as negative control. The seeds were incubated in 10 mL of Buffered Glycerol-complex Medium (BMGY) in a 100 mL shake flask and incubated at 30 °C until the culture reached OD_600_ = 2.0–6.0. The cells were harvested by centrifugation and re-suspended in 50 mL Buffered Methanol-complex Medium (BMMY) and incubated at 30 °C. The methanol induction temperature was set at 30 °C, and 0.7% (*v*/*v*) methanol was fed at 24 h intervals for 5 days. SDS-PAGE was used to observe the expression of the recombinant protein. SDS-PAGE was carried out on a 12% running gel and stained with Coomassie Blue. Cells from the cultures were removed by centrifuging at 6000× *g* for 10 min. The supernatant from mock transformant and transformant containing pPICZαA-*Pc-fps* were concentrated by ultrafiltration using a Millipore set up according to the manufacturer’s instructions with a membrane of 10 kDa cutoff. The supernate from mock transformant and transformant containing pPICZαA-*Pc-fps* was purified by Ni^2+^-chelating chromatography according to the manuals (GE Healthcare, Uppsala, Sweden). The elution buffers were used for further analysis.

### 3.6. Determination of Enzymatic Activity and Product Analysis by GC

The FPS activity of the recombinant protein was measured on the basis of the conversion of DMAPP or GPP with IPP to produce FPP. The FPS activity was assayed according to the method described by previous studies [26]. A reaction mixture for measuring FPS activity contained, in a total volume of 200 μL, 50 mM of MOPS (pH 7.5), 2 mM MgCl_2_, 5 mM of dl-Dithiothreitol, 50 μM of GPP, 50 μM of IPP, and an appropriate amount of the rFPS. The product from a mock purification scheme of transformed colonies contained pPICZαA was set as control. After incubation at 30 °C for 3 h, the mixture was extracted with l-butanol saturated with water and then treated with potato acid phosphatase at 37 °C to hydrolyze the diphosphate moiety. The hydrolyzed products were extracted with hexane and filtered through a 0.22 μm membrane filter, loaded onto a Phenomenex ZB-5MS column, eluted with N_2_ at 1.2 mL/min, detected by Flame Ionization Detector. The oven temperature was first kept constant at 50 °C for 1 min, and then increased to 220 °C at the increment of 10 °C/min, and finally held at this temperature for 10 min. The injector and transfer line temperatures were set at 200 and 250 °C, respectively.

### 3.7. Expression Profile Analysis of Pc-fps under Different Stages of Development 

The mycelia (mycelia were collected after incubation for 5, 10, 15, 20, and 30 days) and primordia were collected for expression profile analysis of *Pc-fps*. The mycelium from the liquid cultures was handled according to the method described by previous study [27]. The real-time quantitative PCR was used to determine the transcripts of the *Pc-fps* under different stages of development. Total RNA was extracted using an RNA Isolation Kit (Omega, Norcross, GA, USA), treated with RNase-free DNase I (TaKaRa, Dalian, China) and then reverse-transcribed to cDNA using an oligo(dT)_17_ primer. Afterwards, the transcriptional level of *Pc-fps* was determined by real-time quantitative PCR. Primers used in the real-time quantitative PCR are presented in the Supplementary Table S1. PCRs were carried out according to the manufacturer’s protocol. After denaturation at 95 °C for 10 min, amplification occurred in three steps: 30 s of denaturing at 95 °C, 35 s of annealing at 55 °C and 30 s of extension at 72 °C for a total of 40 cycles. Transcriptional levels were calculated using the standard-curve method and were normalized against the *P. cocos* 18S rRNA gene as an internal control because its expression was found to be stable under our experimental conditions (data not show). The mycelia at 5 day served as the reference sample and expression of the reference sample was defined as 1.0. The expression of *Pc-fps* (under different stages of development) was defined as the fold of mRNA level over the reference sample. Post real-time quantitative PCR calculations to analyze *Pc-fps* (under different stages of development) were performed according to the 2^−ΔΔ*C*t^ method as described by [28].

### 3.8. Extraction and Analysis of Total Triterpenoids under Different Stages of Development 

The mycelia (mycelia were collected after incubation for 5, 10, 15, 20, and 30 days) and primordia were also collected for analysis of the content of triterpenoids. The triterpenoids of mycelia and primordia were extracted using methods described by previous studies with some modification [22,29]. Freeze-dried mycelia (100 mg) and primordia (100 mg) were extracted for 3 h with 5 mL acetone under sonication at 30 °C for three times. After removing the mycelia and primordia by centrifugation, the supernatants were dried at 60 °C under a vacuum. And then, 0.4 mL 5% vanillin/glacial acetic acid (*w*/*v*) and 1 mL 70% perchloric acid solution were added successively to the tube. The solution was heated for 15 min at 60 °C and then cooled in an ice-water bath to the ambient temperature. The absorbance of the sample was measured at 548 nm after addition of 5 mL glacial acetic acid, with ursolic acid used as the standard.

### 3.9. Analysis of Expression Profile of Pc-fps and Content of Triterpenoids under Methyl Jasmonate (MeJA) Treatments 

*P. cocos* stock and liquid cultures were both maintained on potato dextrose agar (PDA). Seed cultures were grown in 1000 mL flasks containing 200 mL of a PDA and placed on a rotary shaking incubator at 150 rpm at 25 °C for 7 days. The second set of experiments was performed in 250 mL flasks containing 50 mL of PDA after inoculation with 10% (*v*/*v*) of the seed culture. Flasks were then placed on a rotary shaker at 150 rpm at 25 °C. MeJA (Sigma, St. Louis, MO, USA) was dissolved in 100% ethanol and sterilized through 0.22 μm Supor Membrane Acrodisc Syringe Filter (PALL, Cortland, NY, USA). MeJA elicitor solutions were added to the medium on day 0 at four different final concentrations (0, 100, 200 and 300 μM), and three different induction times (24, 48 and 72 h) were tested. Untreated mycelia were used as control. The method for determination of the content of triterpenoids was the same as described above. Semi-quantitative RT-PCR was used to determine the transcripts of the *Pc-fps* gene under MeJA treatment. RNA isolation, DNA digestion, RNA reverse-transcription steps and the RT-PCR amplification conditions were performed as described above. The method for analysis of expression level of *Pc-fps* was also the same as described above, except the reference sample changed as untreated mycelia and expression of untreated mycelia in 24 h was defined as 1.0. 

## 4. Conclusions

In this study, we have isolated *fps* from *P. cocos*, and analyzed the *Pc-fps* promoter and Pc-FPS amino acid sequence. Pc-FPS was expressed in *P. pastoris* and purified. To confirm *Pc-fps* as a functional gene encoding FPS, the purified rPc-FPS was used to catalyze GPP with IPP to produce FPP. After determination of FPP by GC, we verified that the *fps* gene from *P. cocos* is a functional gene encoding FPS. Furthermore, the expression profile of the FPS gene and the content of total triterpenoids at different stages of development and methyl jasmonate treatments were determined. The results indicate that there is a positive correlation between the activity of FPS and the amount of total triterpenoids produced in *P. cocos*. To our knowledge, this is the first report about gene cloning and characterization from *P. cocos* and our result is useful to facilitate the investigation of its structure, expression and the role of FPS in the control of triterpene biosynthesis in *P. cocos*.

## Supplementary Materials

Supplementary table can be found at http://www.mdpi.com/1422-0067/15/12/22188/s1.
